# Supplementary material for: Comparative multiomics analysis of cell physiological state after culture in a basket bioreactor
Source: Sci Rep. 2022 Nov 23;12:20161. doi: 10.1038/s41598-022-24687-4 (PMC9686226; doi:10.1038/s41598-022-24687-4)
Supplement: Supplementary file 1 — Supplementary Information 1. [file 41598_2022_24687_MOESM1_ESM.zip › raw data/Transcriptomic raw data/6.Enrichment/1.GO/TE1vsTC1/DOWN/TE1vsTC1.down_GOdot.pdf]

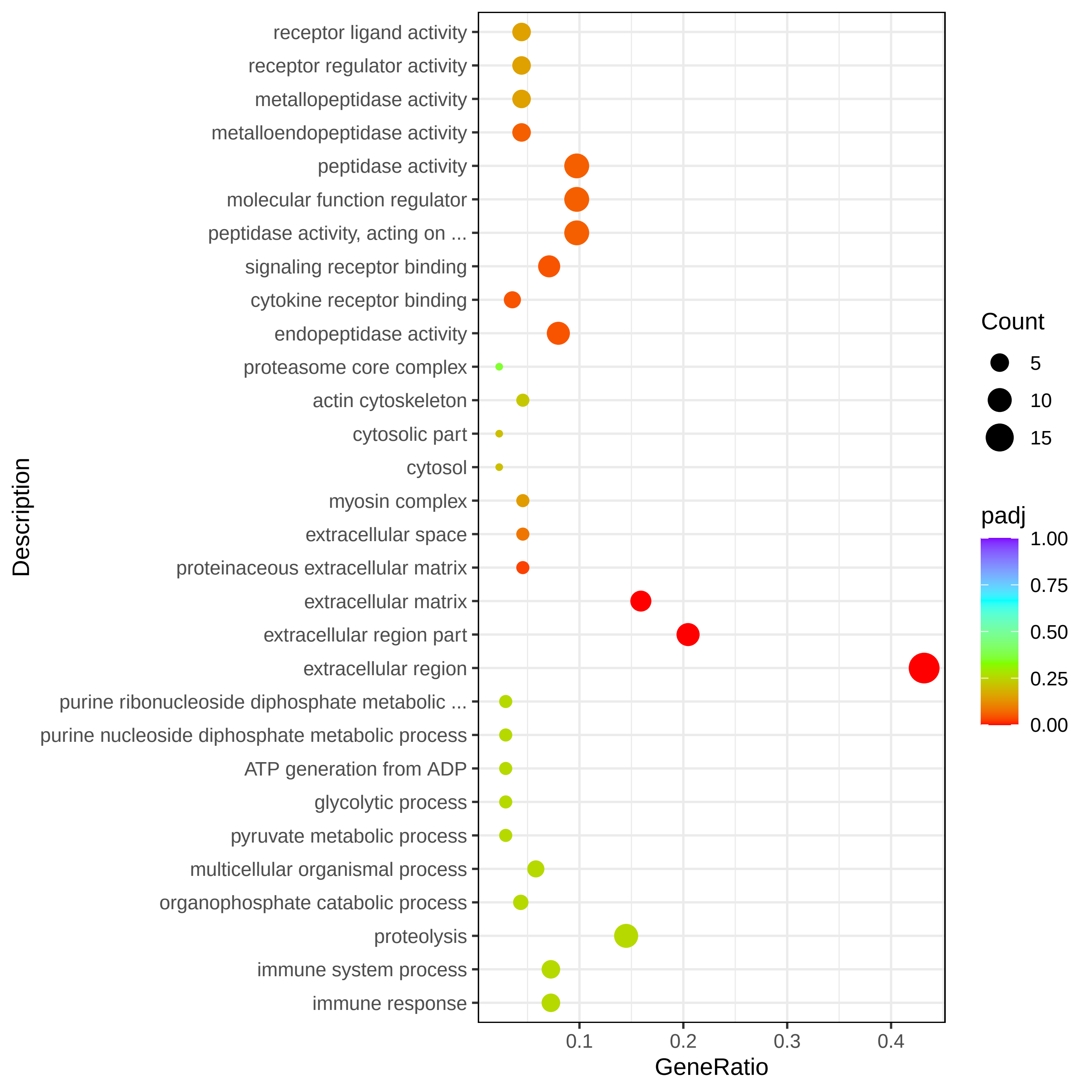
receptor ligand activity
receptor regulator activity
metallopeptidase activity
metalloendopeptidase activity
peptidase activity
molecular function regulator
peptidase activity, acting on ...
signaling receptor binding
cytokine receptor binding
endopeptidase activity
proteasome core complex
actin cytoskeleton
cytosolic part
cytosol
myosin complex
extracellular space
proteinaceous extracellular matrix
extracellular matrix
extracellular region part
extracellular region
purine ribonucleoside diphosphate metabolic ...
purine nucleoside diphosphate metabolic process
ATP generation from ADP
glycolytic process
pyruvate metabolic process
multicellular organismal process
organophosphate catabolic process
proteolysis
immune system process
immune response
Description
0.1
0.2
0.3
0.4
GeneRatio
Count
5
10
15
padj
1.00
0.75
0.50
0.25
0.00
